# A Rare Case of Eosinophilic Granulomatosis with Polyangiitis Associated with Cryoglobulinemia Presenting with a Bullous Skin Eruption of the Lower Limbs

**DOI:** 10.1155/2018/3124281

**Published:** 2018-01-16

**Authors:** D. D. K. Abeyaratne, C. Liyanapathirana, C. L. Fonseka, P. W. M. C. S. B. Wijekoon

**Affiliations:** ^1^University Medical Unit, Colombo South Teaching Hospital, Kalubowila, Sri Lanka; ^2^University Medical Unit, Teaching Hospital Karapitiya, Galle, Sri Lanka; ^3^University of Sri Jayewardenepura, Gangodawila, Nugegoda, Sri Lanka

## Abstract

**Background:**

Eosinophilic granulomatosis with polyangiitis (EGPA) is an antineutrophil cytoplasmic antibody- (ANCA-) associated small vessel vasculitis with multisystem involvement. It is characterized with asthma, eosinophilia, and renal and peripheral nervous system involvement. However, EGPA presenting with bullous skin eruption is an uncommon dermatological manifestation. We report a rare case of EGPA overlapped with mixed essential cryoglobulinemia presenting with a bullous skin eruption.

**Case Presentation:**

A 49-year-old female presented with bilateral lower limb erythematous bullous rash with bilateral lower limb numbness. She had bilateral ankle edema with frothyuria and a recent onset wheeze. Blood investigations revealed a marked peripheral eosinophilia with positive P-ANCA. Skin biopsy was suggestive of leukocytoclastic vasculitis. She also had positive cryoglobulins with a high rheumatoid factor titre. The patient was diagnosed of having EGPA with overlapping mixed essential cryoglobulinemia. Her skin eruptions and systemic manifestations improved with prednisolone and cyclophosphamide therapy.

**Conclusion:**

EGPA can rarely present with a bullous skin eruption and may rarely associate with secondary cryoglobulinemia. Early recognition of these rare manifestations and prompt treatment would prevent further complications and death.

## 1. Introduction

Eosinophilic granulomatosis with polyangiitis (EGPA) is an antineutrophil cytoplasmic antibody- (ANCA-) associated small vessel vasculitis with multisystem involvement. It is a rare primary vasculitis, which typically occurs in patients with a history of recent onset or worsening of bronchial asthma [1]. The clinical features of EGPA typically develop in three sequential phases, although these phases may not always be clearly distinguishable. They are the prodromal phase, eosinophilic phase, and vasculitic phase where organ involvement occurs [2].

There are few cases of EGPA reported in literature where EGPA has been associated with multiple dermatological manifestations, commonest being erythematous macular-papular rash.

Skin manifestations have been reported in 40–70% of the patients with EGPA [3]. They are commonly erythematous macular papules, hemorrhagic lesions ranging from petechiae to extensive ecchymosis, often associated with wheals, necrosis, and ulceration, and cutaneous or subcutaneous nodules that are usually deep seated and tender. They mostly involve extensor surfaces of the upper limbs, particularly elbows and legs [4]. Other less common features include livedo reticularis and papulovesicular rash.

We report a rare case of EGPA presenting as a bilateral lower limb bullous rash, and it was found in an overlap with mixed essential cryoglobulinemia which responded well to glucocorticoids and cyclophosphamide therapy.

## 2. Case Presentation

A 49-year-old previously healthy female got admitted to hospital with a progressively worsening skin rash involving lower limbs for two-week duration. The skin rash has begun as a painful small vesicle on the left foot which enlarged in size, with crops of new vesicles appearing on bilateral lower limbs. Some vesicles enlarged to bullae and then ruptured and healed with scarring.

She also complained of progressive pain and numbness on bilateral lower limbs up to knees, with numbness over left fingers that had progressed over two-week duration. She denied muscle weakness or difficulty in walking. She also had progressive bilateral ankle edema and frothyuria. She denied hematuria or reduced urine output. She had constitutional symptoms with a low-grade fever and generalized malaise. She also gave a history of recent onset wheezing controlled with inhalers but denied cough, sinusitis, or epistaxis. She had no joint symptoms, digital ulcers or gangrene, or gastrointestinal symptoms.

**Figure 1:**
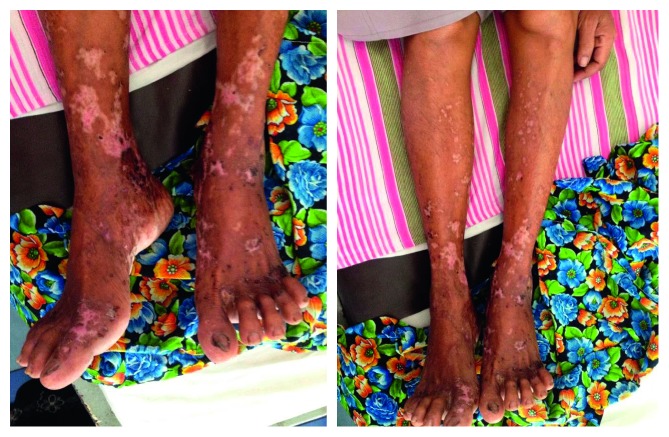
Bilateral lower limb skin rash with ruptured bullae and healed scars.

Lower limb examination revealed an erythematous rash with small blisters and bullae of sizes varying between 2 and 4 cm with hypopigmented healed lesions with marked involvement around the ankle (Figure 1). She also had few small hyperpigmented lesions on the palmar surface of the left hand. She had no lesions in the trunk, face, or mucosa. Neurological examination revealed reduced bilateral lower limb reflexes and sensory impairment up to knee level but had no motor weakness. Joint position sense and vibration sense was intact. Upper limbs did not reveal a demonstrable neurological deficit. All peripheral pulses were felt. She had bilateral pitting ankle edema and had elevated blood pressure 160/100 mmHg. Other system examination revealed normal findings.

Her blood counts revealed a white cell count of 27,000/mm^3^ with a marked eosinophilia of 57% with an absolute count of 15,500/*µ*l. Hemoglobin and platelet count was normal. The blood picture revealed marked eosinophilia without evidence of atypical cells. The erythrocyte sedimentation rate was 54 mm for the 1st hour with an elevated C-reactive protein level of 103 *µ*/l. The urine full report revealed proteinuria with 10–15 red cells without red cell casts. A 24-hour urine protein quantification revealed 1340 mg/24 hr (<150 mg/24 hr) excretion. However, her serum creatinine remained normal. Her serum albumin level was 30 g/l, and there was hypergammaglobulinemia with a total serum globulin level of 49 g/l. Individual globulin levels were not evaluated due to the unavailability of the investigation. Her rheumatoid factor was repeatedly high (512 and 480 *µ*/l) with repeatedly positive serum cryoglobulins in a qualitative assay. Hepatitis B surface antigen (enzyme immunoassay (EIA)) and hepatitis C antibody (EIA) were negative. She had positive P-ANCA (perinuclear and MPO). She had a normal C3 complement level (131.8 mg/dl (88–206 mg/dl)), with low normal C4 complement level (14.4 mg/dl (12–72 mg/dl)). Her other blood investigations, including liver function tests and antinuclear antibody assays, were normal. Chest radiograph and ultrasound abdomen were normal. Her skin biopsy revealed leukocytoclastic vasculitis with few perivascular eosinophilic infiltrates, without granuloma, and her renal biopsy revealed mild interstitial inflammation. Immunofluorescence patterns of the skin biopsy were not assessed.

The patient was diagnosed as having eosinophilic granulomatosis with polyangiitis (EGPA) with mixed essential cryoglobulinemia. The patient was started on a high-dose prednisolone (1 mg/kg) therapy with oral cyclophosphamide. With treatment, she had a marked improvement in her bullous skin rash and constitutional symptoms and inflammatory markers, but she had a residual peripheral neuropathy. The eosinophil count reduced to normal at the 4-week review after discharge, and the steroids were tailed off gradually, after reviewing at 6 weeks. Currently, the patient is followed up on low-dose prednisolone, and the cyclophosphamide was changed to oral azathioprine after achieving complete remission.

## 3. Discussion

Our patient presented with bilateral lower limb skin rash with systemic involvement marked by peripheral neuropathy, proteinuria, and constitutional symptoms. The differential diagnoses considered were a systemic vasculitis, systemic infection, or rarely an internal malignancy with systemic manifestations. However, the patient had typical features of eosinophilic granulomatosis with polyangiitis (EGPA) with marked eosinophilia. The American College of Rheumatologists' criteria for diagnosis include asthma (a history of wheezing or the finding of diffuse high-pitched wheezes on expiration), greater than 10 percent eosinophils on the differential leukocyte count, mononeuropathy (including multiplex) or polyneuropathy, migratory or transient pulmonary opacities on imaging, paranasal sinus abnormality, biopsy containing a blood vessel showing the accumulation of eosinophils in extravascular areas, where four out of six criteria should be fulfilled for the diagnosis [3]. Our patient fulfilled the criteria to arrive at the most likely diagnosis of EGPA. She did not have pulmonary infiltrates on chest radiograph. The patient had positive P-ANCA of MPO variety.

Other than the abovementioned common manifestations, our patient had a different manifestation with an erythematous bullous skin rash starting as vesicles and healing with multiple hypopigmented scars. In literature, there are only very few cases reported on EGPA presented with a bullous rash [3–5]. In one case, bullous rash was associated with marked lung infiltrates on presentation with a negative P-ANCA profile [4]. Two other cases described coexistence of bullous eosinophilic cellulitis which is termed as “Wells syndrome” with EGPA, where the histology from skin biopsy had been eosinophilic without evidence of granuloma or vasculitis unlike in our patient. Wells syndrome, also known as eosinophilic cellulitis, is a rare inflammatory dermatosis of unknown etiology and is commonly seen in adults [6]. Blister formation in bullous pemphigoid has been attributed to the release of major basic protein (MBP) and eosinophil cationic protein (ECP). Similarly, the bullous pemphigoid-like blisters in our case could be due to deposition of eosinophilic cytotoxic proteins in the papillary dermis [5].

Our patient also had associated mixed cryoglobulinemia, which was evidenced by a positive cryoglobulin level with high rheumatoid factor levels. In the literature, there is one reported case of EGPA associated with cryoglobulinemia, but in the presence of hepatitis C infection [7]. In our patient, EGPA may have associated with a secondary cryoglobulinemia which has occurred in association with an autoimmune disease. She had negative hepatitis B and C serology.

Management of ANCA-associated vasculitis includes rapid diagnosis and early initiation of immunosuppressants. Our patient was started on oral cyclophosphamide and high-dose prednisolone (1 mg/kg) where she had a marked response within first few weeks.

## 4. Conclusion

EGPA is an ANCA-associated vasculitis with multiorgan involvement. EGPA presenting as a bullous eruption is a rare manifestation in literature. Association of EGPA with possibly secondary cryoglobulinemia in the absence of hepatitis infection is a unique association that we observed in our case.
